# NQO1 Is Regulated by PTEN in Glioblastoma, Mediating Cell Proliferation and Oxidative Stress

**DOI:** 10.1155/2018/9146528

**Published:** 2018-11-25

**Authors:** Shilin Luo, Kecheng Lei, Daxiong Xiang, Keqiang Ye

**Affiliations:** ^1^Department of Pathology and Laboratory Medicine, Emory University School of Medicine, Atlanta, GA 30322, USA; ^2^Department of Pharmacy, the Second Xiangya Hospital, Central South University, Changsha, Hunan 410011, China

## Abstract

Glioblastoma multiforme (GBM) is a highly aggressive brain tumor with a dismal prognosis, and the patients carrying EGFR-driven tumors with PTEN mutation do not respond to anti-EGFR therapy. The molecular mechanisms for this resistance remain unknown. Here, we show that PTEN induces the expression of NQO1, a flavoenzyme with dual roles in pro- and antitumorigenesis that decreases the formation of reactive oxygen species (ROS), which mediates the oxidative stress and GBM cell proliferation. NQO1 is reduced in EGFRvIII-overexpressed U87MG cells associated with low ROS, whereas NQO1 is highly escalated in PTEN stably expressed U87MG/EGFRvIII cells with high ROS. Interestingly, knockdown of NQO1 augments ROS and diminishes cell proliferation. Conversely, overexpression of NQO1 attenuates ROS and increases cell proliferation. By contrast, overexpression of PINK1, a PTEN-induced kinase 1, represses ROS and inhibits GBM cell proliferation. Therefore, our findings support that NQO1 displays a paradoxical role in mediating GBM growth in response to tumor suppressor PTEN.

## 1. Introduction

Glioblastoma multiforme (GBM) is the most malignant human brain tumor. It is highly aggressive, infiltrative, and destructive. In clinical trials of radiation therapy and temozolomide chemotherapy following surgical resection, the average survival period for the patient is around 60–70 weeks [1]. Specific therapeutic targeting of GBM subclasses remains a goal in neurooncology. The key features of primary GBM include amplification of epidermal growth factor receptor (EGFR) activity, deletion or mutation of homozygous cyclin-dependent kinase (CDK) inhibitor p16INK4A (CDKN2A), alterations in phosphatase and tensin homolog (PTEN) on chromosome 10, and deletion of INK4a [2]. As a receptor tyrosine kinase (RTK), EGFR mediates cell growth and proliferation via downstream effectors such as Ras and PI-3-Kinase (PI3K) and is regulated by tumor suppressor genes NF1 and PTEN. PTEN, a protein implicated in various cellular processes including metabolism, apoptosis, cell proliferation, and survival, suppresses the PI3K/Akt pathway via dephosphorylating PIP_3_ (phosphatidyl-3,4,5-triphosphate) into PIP_2_ (phosphatidyl-4,5-diphosphate). One of the most selective genetic alterations in GBM is the amplification of EGFR, which occurs in approximately 40% of GBMs. Either wild-type or mutated forms of EGFR can be amplified. The most common mutated form lacks exons 2–7, resulting in constitutively active tyrosine kinase activity (EGFRvIII) [3]. In clinical trials, patients carrying EGFR-driven tumors with PTEN mutation do not respond to anti-EGFR treatment, but the molecular mechanisms for this resistance remain unknown [4].

Amplification of EGFR activity or its constitutive activation due to truncation, PTEN mutation, and loss of chromosome 10 is found in primary GBM tumors, while TP53 mutations are common in secondary GBM [5, 6]. These mutations affect the redox balance in the cancer cells. For instance, EGFR activation by EGF induces endogenous production of intracellular reactive oxygen species (ROS) and H_2_O_2_ in cancer cell lines [7, 8]. Upon ligand binding, EGFR forms homo- and heterodimers that activate several intracellular signal pathways, such as PI3K/Akt and Ras/mitogen-activated protein kinase (MAPK), resulting in DNA synthesis augmentation [7]. High doses of H_2_O_2_ (200 pM) escalate EGFR Tyr autophosphorylation, leading to generation of ROS [7]. In acting as a tumor suppressor, PTEN negatively regulates the PI3K/Akt pathway via hydrolyzing the key second messenger PI-(3,4,5)P_3_ [9, 10]. PTEN is also regulated by redox status, specifically by H_2_O_2_, which can trigger a disulfide bond formation between Cys71 and Cys124 in the phosphatase domain [11], altering its interaction with signaling and regulatory proteins [11, 12]. Presumably, overexpression of EGFR may increase H_2_O_2_ levels, disturbing a number of signaling pathways and stimulating cell survival and proliferation.

NAD (P)H: quinone oxidoreductase (NQO1, also called as DT-diaphorase) is a cytosolic flavoenzyme that is crucial in protecting against endogenous and exogenous quinones via catalyzing two- or four-electron reductions of the substrates [13]. NQO1 possesses multiple enzymatic and nonenzymatic functions. For instance, NQO1 has superoxide scavenging activity, stabilizing p53 and other 20S proteasome-degradable tumor suppressor proteins [14]. NQO1 occurs in all tissues with the highest expression levels in epithelial, vascular endothelial, adipocytes, and cancer cells, especially liver tumors [15]. NQO1 gene expression is mainly regulated by the ARE (antioxidant response element) under both normal and oxidative stress conditions [16]. The NQO1 gene contains ARE in its promoter region and is regulated by the nuclear factor (erythroid-derived)-like 2 (Nrf2) [17]. Xenobiotics, antioxidants, oxidants, UV light, and ionizing radiations mediate NQO1 expression via Keap1/Nrf2/ARE pathway [18]. Interestingly, two polymorphic forms of NQO1 that reduce cellular NQO1 activity are associated with increased risk of cancers [19–21]. Although a lowered or absent NQO1 activity is correlated with increased susceptibility for human cancer development [19, 22], several studies reveal that NQO1 is upregulated in a number of cancers [23–25]. Consequently, identification of high affinity and selective inhibitors of NQO1 might be an attractive strategy for treating cancers.

In the current study, we provide innovative evidence demonstrating that NQO1 acts as a downstream target of PTEN in glioblastoma cells, promoting GBM cell proliferation and suppressing ROS. In alignment with its paradoxical roles as both anticancer enzyme and oncogene, NQO1 augments GBM cell growth in response to PTEN expression, which is in sharp contrast to another downstream target of PTEN, PINK1, which also possesses antioxidant activity. This interesting finding might provide insight into the molecular mechanism of why gliomas with EGFR amplification and PTEN deletion are resistant to anti-EGFR therapy.

## 2. Materials and Methods

### 2.1. Cell Lines and Reagents

The human glioblastoma cell line U87MG was stably transfected with vector control, PTEN, EGFR, EGFR/PTEN, EGFRvIII (Y1068F), and EGFRvIII/PTEN, which were cultivated in DMEM with 10% FBS and 1% pen/strep/glutamine supplemented with various selection antibiotics. For wild-type EGFR, 0.7 *μ*g/mL of puromycin was included; for EGFRvIII, 150 *μ*g/mL of hygromycin was employed; and for PTEN, 400 *μ*g/mL of G418 was added. LN229 and LN229/EGFR were maintained in DMEM with 10% FBS and 150 *μ*g/mL of hygromycin was added in the selecting process of LN229/EGFR cells. Cultures were maintained in 5% CO_2_ and air humidified in a 37°C incubator. The horseradish peroxidase-linked immunoglobulin G (lgG) secondary antibody was obtained from GE Healthcare. EGF was purchased from Sigma-Aldrich (#E9644). All antibodies were purchased from Cell Signaling Technology or Santa Cruz Biotechnology.

### 2.2. Western Blotting

Western blotting was performed using a standard protocol. To prepare whole cell lysates, cells were lysed with lysate buffer (50 mM Tris pH 7.4, 150 mM NaCl, 1 mM EDTA, 1% Triton-X 100, 50 mM NaF, 10 mM sodium pyrophosphate, and 10 mM Sodium glycerophosphate). Nuclear fractions were prepared using a Nuclear Extraction Kit (#78833, Thermo) according to the manufacturer's protocol. Protein concentration was measured by Bradford assay, and the extracts were stored at −80°C until analyzed. Equal amount of protein (20–50 *μ*g) was loaded for blotting with indicated specific antibodies.

### 2.3. ROS Staining Assay

Cells were plated onto coverslips and fixed with 4% paraformaldehyde for 10 min. Cells were firstly washed twice with Hank's balanced salt solution containing Ca^2+^/Mg^2+^, then added with 5 *μ*M CM-H_2_DCFDA (ROS dye, #C6827, Invitrogen) that was diluted in PBS for 1 h at 37°C. ROS species contain hydrogen peroxide, superoxide anion, and hydroxyl radical were detected by CM-H_2_DCFDA. Then, using wash buffer to wash three times and allowed to recover for 10 min at 37°C. Confocal microscope was employed to obtain the images.

### 2.4. Protein Carbonyl Assay

Protein carbonyls were measured from cell homogenates using 2,4-dinitrophenylhydrazine derivatization according to the manufacturer's instructions of Protein Carbonyl Assay Kit (#ab126287, Abcam, Cambridge MA).

### 2.5. GSH/GSSG Analysis

GSH/GSSG ratio in various U87MG cell lines was determined using GSH/GSSG Ratio Detection Assay Kit (Fluorometric-Green) (#ab138881, Abcam), according to the manufacturer's guidelines. In brief, the whole cell lysates were diluted to 1 : 80 for GSH analysis, serial dilution of GSH and GSSG stock standards were prepared as standards. A one-step fluorimetric reaction of samples with respective assay buffer and probes were incubated for 30 min. Then, fluorescence intensity was monitored at EX/EM of 490/520 nm.

### 2.6. Transfection of siRNA and Plasmids

The NQO1-siRNA (#sc-37139), PTEN-siRNA (#sc-29459), and nontargeting siRNA (#sc-37007) as a control were purchased from Santa Cruz Biotechnology. GFP-PTEN (#13039) plasmid was purchased from Addgene. GBM cells were transfected with 20 nM siRNA or 2 *μ*g plasmid using the Lipofectamine 3000 and P3000 (#L3000075, Invitrogen) according to the manufacture's protocol with opti-MEM (#31985070, Gibo) as a transfection solution. Treated cells were incubated at 37°C for 48 h and then harvested for further experiments.

### 2.7. In Vitro Cell Proliferation Assay

Cell proliferation was monitored by MTT incorporation (Cell Signaling, Beverly, MA) assay. To analyze the proliferation of human GBM under the effect of transfection, cells were collected after 24 h transfection, and then approximately 3000 cells were seeded into a 96-well plate in triplicate, which were incubated at 37°C for indicated time. 5 *μ*L of MTT (3 mg/mL) solution was added to each well when the time was up. After continuing to incubate for 3 h, the supernatant was removed, and then 100 *μ*L of dimethyl sulfoxide was added into each well and mixed thoroughly. Finally, to ensure all crystals were totally dissolved after 10 min of shaking, the optical density (OD) was determined at 490 nm by the microplate reader (BioTek Instruments Inc.). The cell viability assay was repeated at least twice.

### 2.8. Statistics Analysis

Data are presented as mean ± s.e.m. Statistical evaluation was carried out by Student's *t*-test or ANOVA followed by Tukey's multiple comparison tests versus controls. Data were considered statistically significant when ^∗^ *P* < 0.05 and ^∗∗^ *P* < 0.01. All statistical analysis was performed by the program Prism (GraphPad Software, La Jolla, CA, USA).

## 3. Results

### 3.1. NQO1 Is Coupled with PTEN Expression and Oxidative Stress Levels in GBM Cells

**Figure 1:**
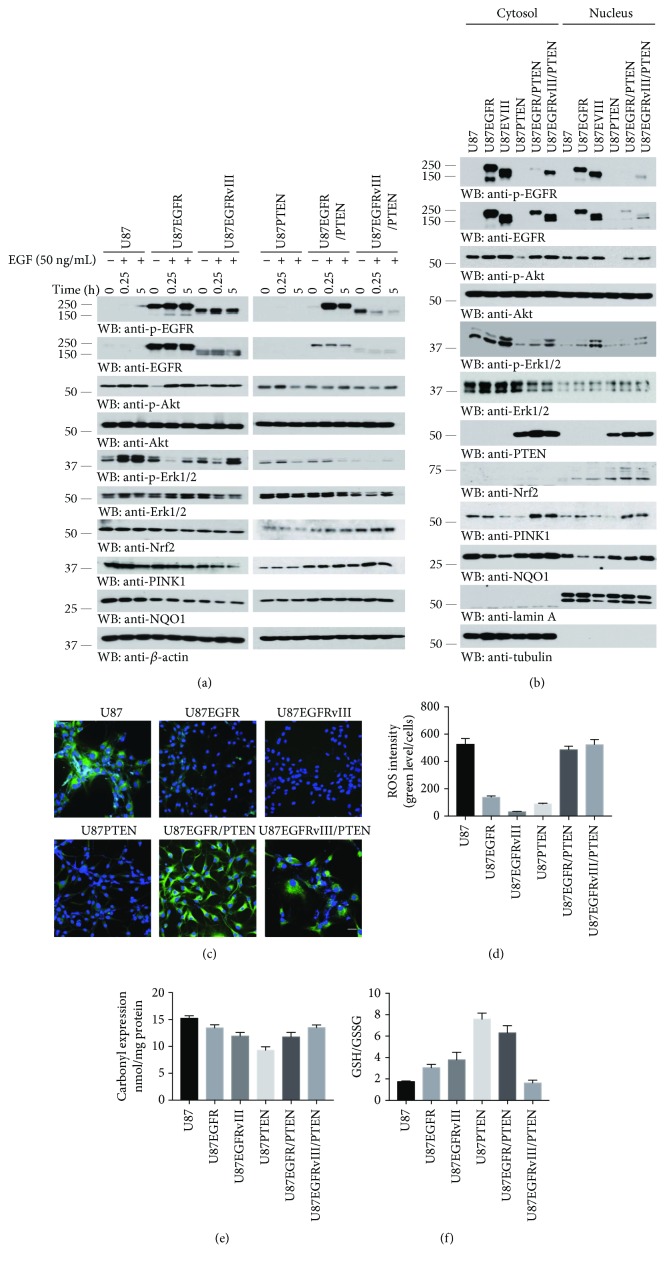
PTEN upregulates NQO1 expression and oxidative stress levels in various U87MG GBM cells. (a) EGFR regulates NQO1 expression in a PTEN-dependent manner. Various U87MG cells were treated with EGF (50 ng/mL) for the indicated time after serum starvation overnight. EGFR signaling pathways were analyzed by immunoblotting with the indicated antibodies. (b) Western blot analysis of EGFR signaling pathways in the cytosolic and nuclear fractions of various U87MG cells without EGF stimulation. Tubulin and Lamin A were employed as loading control proteins for cytosolic and nuclear fractions, respectively. Western blots are representative of three independent experiments. (c) Representative images of ROS levels in various U87MG cells. ROS-positive cells were detected by the indicator dye CM-H_2_DCFDA. Scale bar, 50 *μ*m. (d) Quantification of ROS intensities using the ratio of fluorescent intensity and cell numbers. Data represent mean ± s.e.m. (*n* = 5). Protein carbonyl expression (e) and GSH/GSSG ratio (f) analysis for whole cell lysates of various U87MG cells (mean ± s.e.m., *n* = 3).

U87MG GMB cells express wild-type EGFR with PTEN deletion. Our previous study shows that EGFRvIII stably transfected U87MG cells display hyperproliferation, mimicking the highly malignant primary gliomas with EGFR mutation and amplification [26, 27]. As expected, EGFR was greatly phosphorylated in both U87MG/EGFR and U87MG/EGFRvIII cells even under basal levels as compared to U87MG cells. Acute or sustained EGF stimulation barely affected EGFR activation status in U87MG/EGFR and U87MG/EGFRvIII cells. By contrast, EGF robustly stimulated p-EGFR in U87MG/EGFR/PTEN cells, whereas p-EGFR signals faded away after EGF treatment in U87MG/EGFRvIII/PTEN cells (Figure 1(a), top and 2^nd^ panels). Accordingly, the downstream p-Akt and p-Erks temporally oscillated in response to EGF in U87MG cells, and the signals were significantly reduced in U87MG/PTEN cells. The similar observations occurred in other cells with EGFR or PTEN stable transfection (Figure 1(a), 3^rd^–6^th^ panels). Interestingly, NQO1 was evidently reduced in EGFR or EGFRvIII cells as compared to the parental U87MG cells. In contrast, its expression levels were escalated in the same cells in the presence of PTEN (Figure 1(a), 9^th^ panel). Noticeably, its transcription factor Nrf2 revealed the similar pattern. Interestingly, PINK1, a PTEN-induced putative kinase 1, displayed the same expression manner as NQO1 (Figure 1(a), 8^th^ and 9^th^ panels). Since Nrf2 nuclear translocation dictates its transcription activity, we performed subcellular fractionation. Remarkably, Nrf2 was selectively demonstrable in the nuclear fraction, and EGFR and EGFRvIII increased its amount, which was further augmented by PTEN. Consequently, both NQO1 and PINK1 expression levels were higher in cells with PTEN expression (Figure 1(b)). To further confirm this phenomenon, we tested the expression levels of target proteins in LN229 and LN229/EGFR stable GBM cells expressing intact PTEN. In response to EGF, p-EGFR signals in LN229/EGFR cells were greatly elevated at 0.25 h and the robust activation was detectable even at 5 h. Consequently, the downstream p-Akt and p-Erk activities were stronger than the parental LN229 cells (Supplementary Figure 1a, top–6^th^ panels). Nevertheless, Nrf2 and PINK1 were about comparable between these two cell lines regardless of EGF stimulation (Supplementary Figure 1a, 8^th^ and 9^th^ panels). It was very interesting that PTEN levels appear higher in LN229/EGFR cells than the parental cells, together with NQO1 expression levels more abundant in LN229/EGFR cells (Supplementary Figure 1a, 7^th^ and 10^th^ panels).

To explore whether the ROS levels are correlated with the expression of antioxidant enzyme NQO1, we examined ROS production using the fluorescent probe chloromethyl derivative of H_2_DCFDA (CM_2_-H_2_DCFDA). A robust diminishment in ROS production was observed with EGFR or EGFRvIII expression in U87MG cells as compared with the parental U87MG, and PTEN strongly flipped the ROS status in U87MG cells (Figure 1(c)). Quantitative analysis of ROS intensities mirrored the discoveries (Figure 1(d)), indicating that oxidative stress is much higher in U87MG and U87MG/EGFRvIII/PTEN cells than in four other U87MG cell lines. Additional indicators of oxidative stress including protein carbonyls expression and GSH/GSSG ratio analysis also validated this finding (Figures 1(e) and 1(f)). ROS and NQO1 costaining was performed on different U87MG cells (Supplementary Figure 1d). Fitting with the immunoblotting of expression levels of NQO1, the positive relationship between NQO1 and ROS levels suggested the intrinsic correlation. The similar oxidative stress trends were observed in LN229 and LN229EGFR cells (Supplementary Figure 1b and 1c). Hence, PTEN manipulates NQO1 expression, correlating with the oxidative stress in GBM cells.

### 3.2. NQO1 Deletion Increases ROS and Diminishes U87MG/EGFRvIII Cell Proliferation

**Figure 2:**
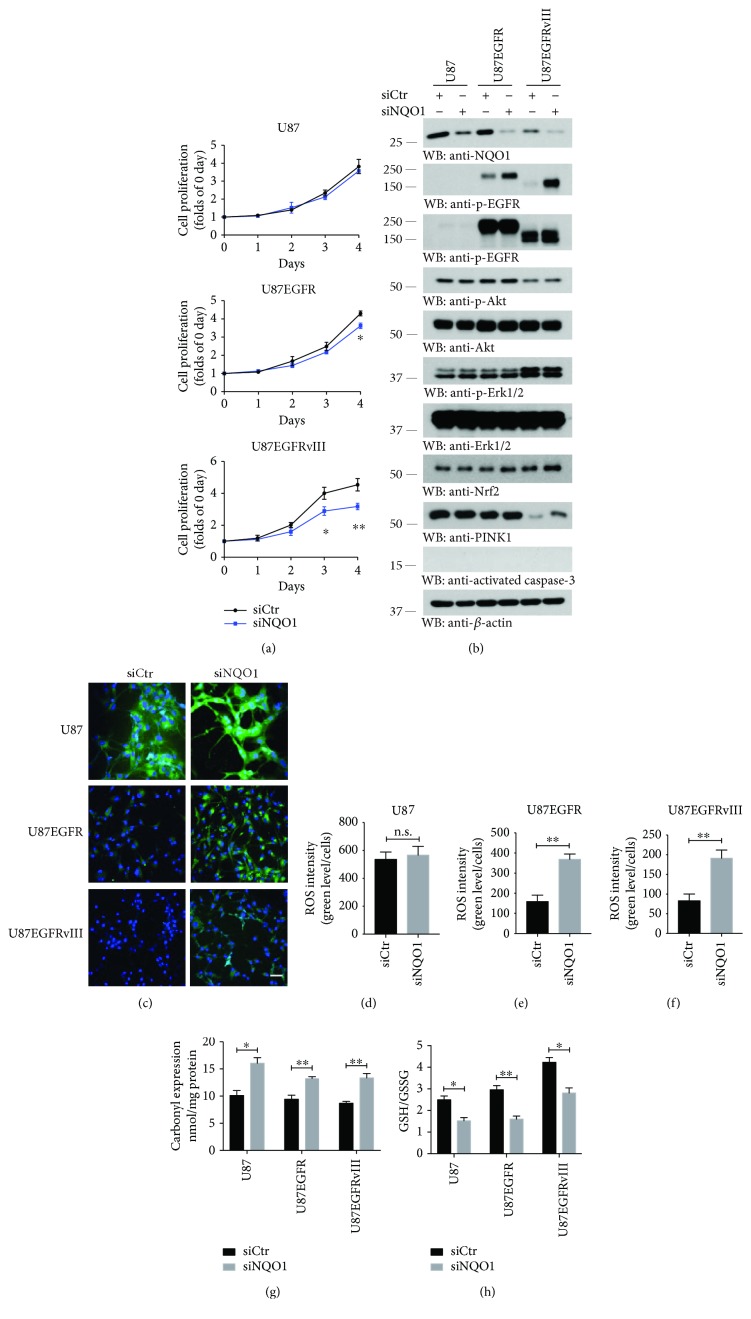
NQO1 knockdown diminishes U87MG/EGFRvIII cell proliferation. (a) Cell growth assay. Proliferation of U87MG cells without PTEN expression transfected with si-control or NQO1 siRNA for up to 4 days. Data are mean ± s.e.m. (*n* = 3; ^∗^ *P* < 0.05, ^∗∗^ *P* < 0.01; Student's *t*-test). (b) Depletion of NQO1 increases the expression Nrf2 and PINK1 in U87MG/EGFRvIII cells. Western blot analysis of EGFR signaling pathways after NQO1 knockdown. (c) ROS staining by the indicator dye CM-H_2_DCFDA for NQO1 knockdown compared with si-control in various U87MG cells. Scale bar, 50 *μ*m. (d–f) Analysis of ROS positive cells in (c). Data represent mean ± s.e.m. of five independent images (n.s. means not statistically significant, ^∗∗^ *P* < 0.01; Student's *t*-test). Protein carbonyl expression (g) and GSH/GSSG ratio (h) analysis after NQO1 knockdown (*n* = 3; ^∗^ *P* < 0.05, ^∗∗^ *P* < 0.01; Student's *t*-test). Western blot data are representative of three independent experiments.

PTEN significantly represses U87MG cell proliferation [28]. To investigate the role of NQO1 in U87MG proliferation, we knocked down this enzyme using its specific siRNA. In a cell proliferation assay, depletion of this gene prominently reduced U87MG/EGFRvIII cell growth, followed by U87MG/EGFR cells. Knockdown did not obviously affect U87MG cell proliferation (Figure 2(a)). Notably, knockdown of NQO1 escalated p-EGFR signals in both U87MG/EGFRvIII and EGFR cells without affecting the downstream p-Akt or p-Erks but somehow elevated Nrf2 and PINK1 levels in both U87MG/EGFR and U87MG/EGFRvIII cells. NQO1 knockdown suppression of cell proliferation was not due to cell death, because caspase-3 was not activated in any of U87MG cells (Figure 2(b)). As expected, elimination of NQO1 augmented ROS levels in U87MG cells by 2–3-fold relative to both EGFR and EGFRvIII cells (Figures 2(c)–2(f)). Protein carbonyl expression and GSH/GSSG ratio analyses confirmed these findings (Figures 2(g) and 2(h)). We made the same observations in PTEN stably transfected U87MG cells (Supplementary Figure 2). Thus, elimination of NQO1 substantially augments the oxidative stress in U87MG cells.

### 3.3. NQO1 Knockdown Decreases LN229 GBM Cell Proliferation without Altering ROS

**Figure 3:**
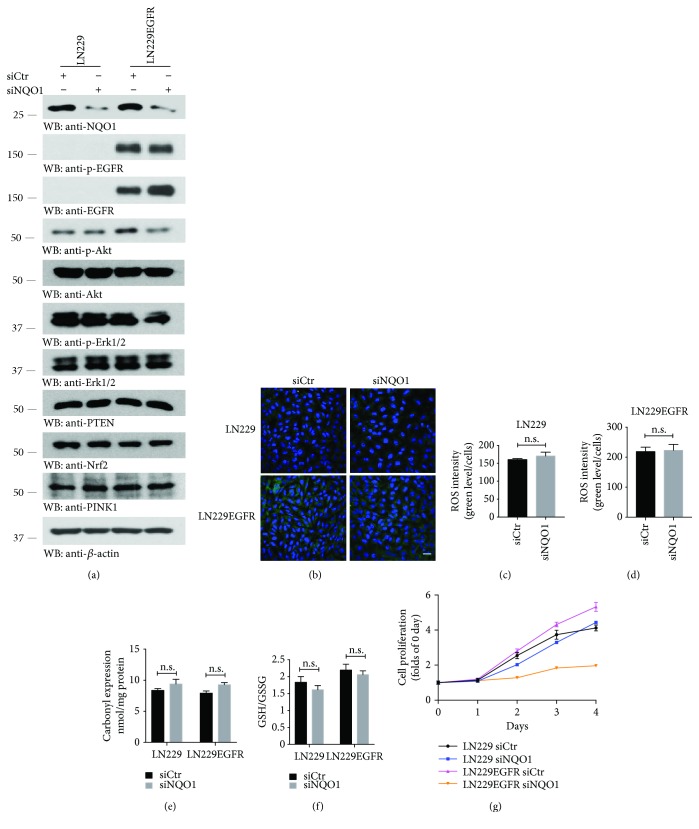
Knocking down NQO1 decreases cell proliferation of LN229 GBM. (a) NQO1 knockdown decreases the expression of p-Akt and p-Erks without affecting Nrf2 and PINK1 in LN229/EGFR cells. Western blot analysis of EGFR signaling pathways after NQO1 knockdown in LN229 cell lines. (b–f) NQO1 deletion does not affect the oxidative stress in LN229 cells. Oxidative stress indicators, the levels for ROS (b–d) and protein carbonyl expression (e) as well as GSH/GSSG ratio (f) analyses followed after NQO1 knockdown. Data represent mean ± s.e.m. (*n* = 3, n.s. means not statistically significant; Student's *t*-test). Scale bar, 50 *μ*m. (g) Cell proliferation assays. NQO1 knockdown decreases LN229 cell proliferation. The proliferation of LN229 cells with PTEN intact transfected with si-control or NQO1 siRNA for up to 4 days. Data are mean ± s.e.m. (*n* = 3 experiments). Western blot data are representative of three independent experiments.

To further investigate the roles of NQO1 in regulating cell proliferation and oxidative stress of LN229 GBM cells with PTEN intact, we did the same experiments as those for U87MG cells. Unlike U87MG cells, knockdown of NQO1 did not affect p-EGFR activities in LN229/EGFR cells, though it decreased p-Akt and p-Erks levels as compared to si-control in LN229/EGFR. However, PTEN, Nrf2, or PINK1 remained intact in both cell lines regardless of NQO1 depletion (Figure 3(a)). The analyses for ROS fluorescent probe staining, protein oxidation, and GSH/GSSG ratio indicated that NQO1 depletion had no obvious effect on the oxidative status in either cell line (Figures 3(b)–3(f)). Remarkably, knockdown of NQO1 specifically decreased LN229/EGFR cell proliferation without affecting LN229 cells (Figure 3(g)). Therefore, these studies suggest that NQO1 depletion suppresses EGFR stably transfected LN229 GBM cell growth without altering the ROS levels.

### 3.4. NQO1 Overexpression Represses ROS and Elevates GBM Cell Growth

**Figure 4:**
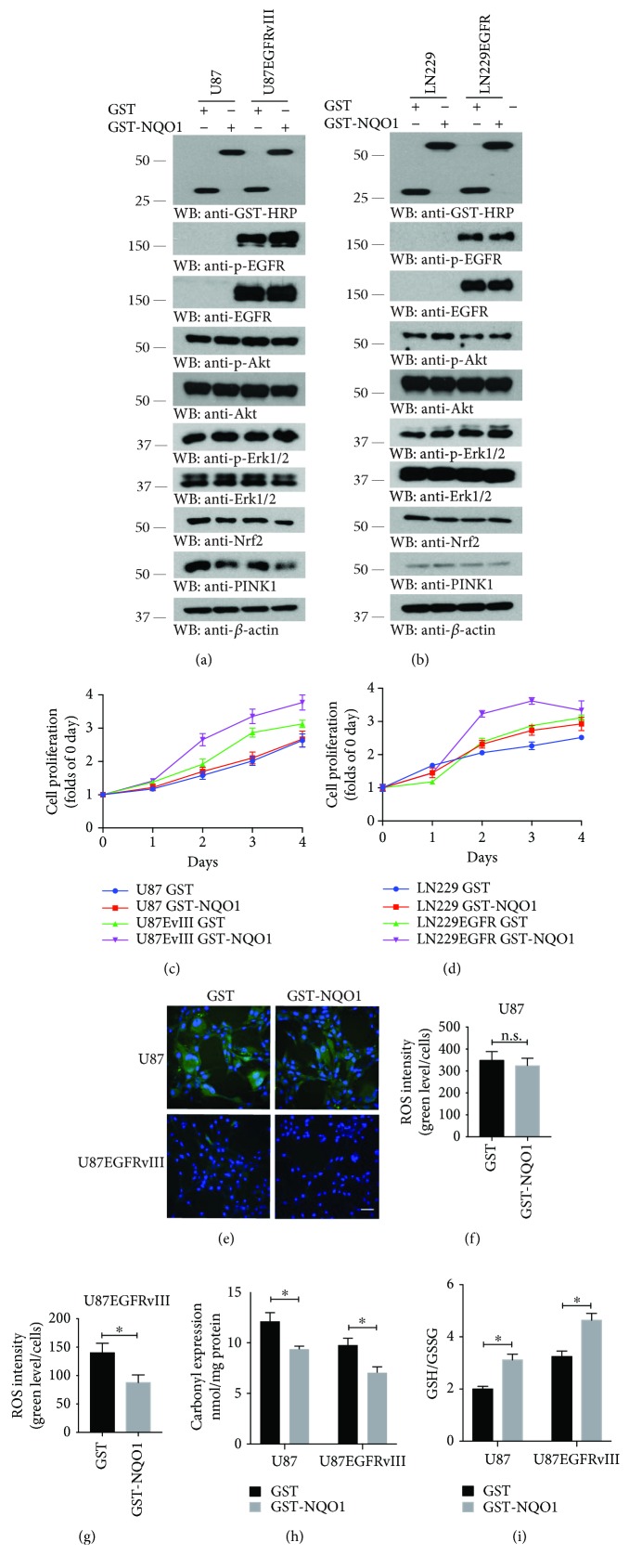
NQO1 overexpression augments the proliferation of GBM cells. (a, b) NQO1 overexpression represses PINK1 in U87MG cells without affecting its expression in LN229 GBM cells. Western blot analysis of EGFR signaling pathways after transfection with GST or GST-NQO1 in U87MG and U87MG/EGFRvIII, and LN229 and LN229/EGFR cells, respectively. (c, d) NQO1 overexpression strongly elevates proliferation of U87MG/EGFRvIII and LN229/EGFR cells. Cells were transfected with GST or GST-NQO1 for up to 4 days. Data represent mean ± s.e.m. (*n* = 3 experiments). (e) Representative images of ROS staining. CM-H_2_DCFDA staining assay showed that NQO1 overexpression repressed ROS levels in U87MG/EGFRvIII but not in U87MG cells. Scale bar, 50 *μ*m. (f, g) Quantification of ROS positive cells in (e). Data represent mean ± s.e.m. (*n* = 5; n.s. means not statistically significant, ^∗^ *P* < 0.05; Student's *t*-test). Protein carbonyl expression (h) and GSH/GSSG (i) assays indicate NQO1 overexpression downregulates the oxidative stress in both U87MG and U87MG/EGFRvIII cells (mean ± s.e.m.; *n* = 3; ^∗^ *P* < 0.05; Student's *t*-test). Western blot data in (a) and (b) are representative of three independent experiments.

To interrogate in-depth the cell proliferation and ROS status relationship by NQO1, we overexpressed NQO1 in PTEN-deficient U87MG and U87MG/EGFRvIII cells. Immunoblotting analysis revealed that NQO1 did not affect p-EGFR or p-Akt activities. Noticeably, both Nrf2 and PINK1 were attenuated when NQO1 was highly expressed (Figure 4(a)). Nonetheless, in PTEN intact LN229 or LN229/EGFR cells, overexpression of NQO1 did not affect any of these events (Figure 4(b)). Cell proliferation assay showed that GST-NQO1 overtly augmented U87MG/EGFRvIII cell growth, but it did not alter U87MG cell growth. On the other hand, GST-NQO1 increased both LN229 and LN229/EGFR cell proliferation as compared to GST control (Figures 4(c) and 4(d)). Oxidative stress analysis demonstrated that GST-NQO1 significantly repressed the ROS levels in U87MG/EGFRvIII cells without affecting U87MG cells (Figures 4(e)–4(i)). By contrast, it did not influence ROS status in either LN229 or LN229/EGFR cells (Supplementary Figure 3). Therefore, these data suggest that NQO1 increases GBM cell proliferation independent of ROS suppression.

### 3.5. PTEN Mediates NQO1 Expression in LN229 GBM Cells

**Figure 5:**
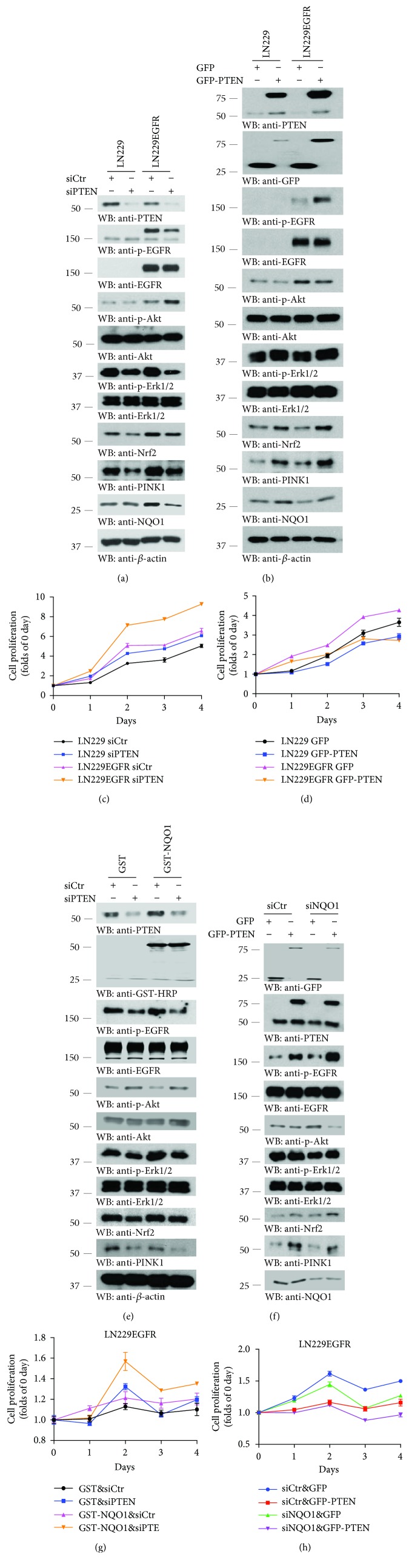
PTEN upregulates NQO1 expression and inhibits cell proliferation of LN229 GBM cells. (a, b) PTEN regulates NQO1 expression. The protein expression profiles of EGFR signaling pathways in LN229 GBM cells after the transfection with PTEN siRNA and GST-PTEN, respectively. Data are representative of three independent experiments. (c, d) PTEN mediates cell proliferation of LN229 GBM cells. Cell proliferation was monitored after the transfection with si-control or si-PTEN (c) and GFP or GFP-PTEN (d) for up to 4 days, respectively. Data represent mean ± s.e.m. from three experiments. (e, f) Western blot analysis of protein expression of LN229/EGFR cells cotransfected with the combination of si-control/PTEN siRNA and GST/GST-NQO1. (g, h) NQO1 and PTEN coregulate cell proliferation of LN229 GBM cells. LN229/EGFR cells were transfected with indicated conditions for up to 4 days. Data represent mean ± s.e.m. (*n* = 3 experiments).

To assess whether PTEN plays any role in mediating NQO1 expression in LN229 cells, we knocked down PTEN with its specific siRNA. Interestingly, depletion of PTEN reduced p-EGFR in LN229/EGFR cells. However, p-Akt signals in these cells were elevated, fitting with the fact that PI3K inhibitor PTEN was eliminated (Figure 5(a), top–5^th^ panels). It is worth noting that NQO1 in LN229/EGFR cells was evidently attenuated, so was its upstream transcription factor Nrf2 (Figure 5(a), 8^th^ panel). As a downstream effector of PTEN, PINK1 was decreased when PTEN was knocked down (Figure 5(a), 7^th^ panel). On the other hand, overexpression of PTEN augmented p-EGFR in LN229/EGFR cells. Consequently, p-Akt was repressed when PTEN was overexpressed (Figure 5(b), top–6^th^ panels). Once more, NQO1 was escalated when PTEN was higher, so were both Nrf2 and PINK1 (Figure 5(b), 9^th^–11^th^ panels). In alignment with the tumor suppressive functions of PTEN, cell growth rates were increased when PTEN was knocked down, whereas cell proliferation was attenuated while PTEN was overexpressed (Figures 5(c) and 5(d)).

To dissect the molecular relationship between NQO1 and PTEN, we transfected GST-NQO1 into LN229/EGFR cells in the presence of control siRNA or si-PTEN. Interestingly, depletion of PTEN decreased p-EGFR no matter whether GST or GST-NQO1 was transfected. On the contrary, p-Akt was elevated. Again, PINK1 was ameliorated when PTEN was depleted (Figure 5(e)). Cell proliferation assay indicated that GST-NQO1 increased cell growth compared with GST control, and it was further escalated when PTEN was knocked down (Figure 5(g)).

Next, we switched the experiments in LN229/EGFR cells with PTEN overexpression in the presence of control si-RNA or si-NQO1. Immunoblotting indicated that p-EGFR was prominently elevated when GFP-PTEN was overexpressed, no matter whether NQO1 was eradicated or not (Figure 5(f)). Either depletion of NQO1 or overexpression of PTEN reduced cell proliferation, and the peak effect was observed when PTEN was overexpressed and NQO1 was depleted at the same time in LN229/EGFR cells (Figure 5(h)).

### 3.6. PINK Overexpression Decreases Proliferation and ROS Levels of U87EGFRvIII Cells

**Figure 6:**
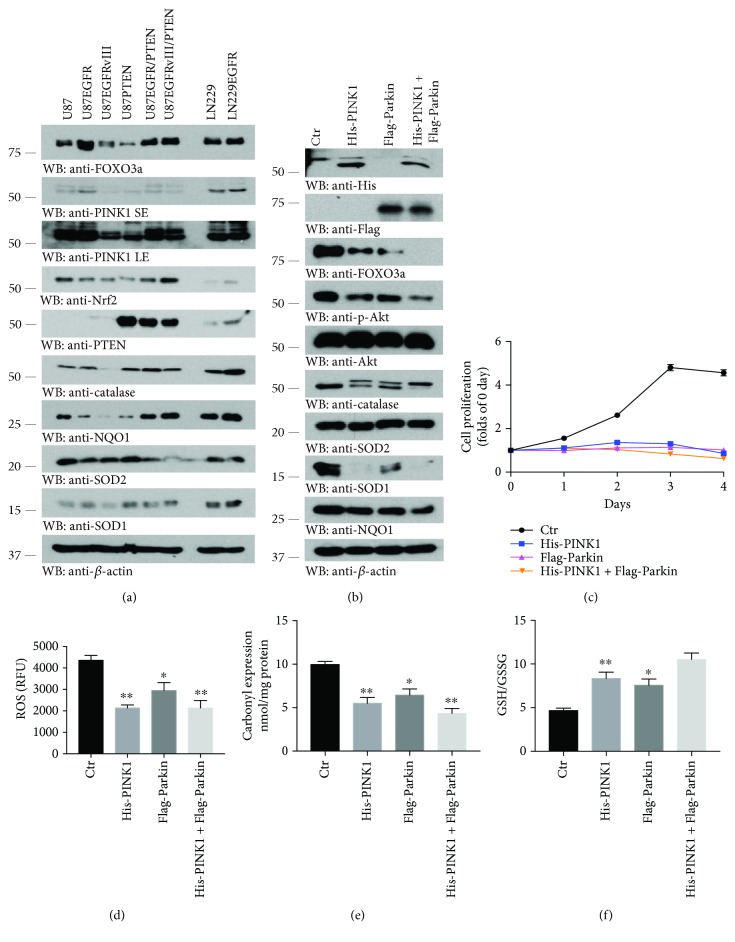
Overexpressing PINK1 attenuates proliferation and ROS levels of U87EGFRvIII cells. (a) The protein expression profiles of the antioxidant system of various GBM cells. (b) Transfection with PINK1, Parkin, or both of them attenuated expression of the majority of antioxidant proteins except SOD2 in U87MG/EGFRvIII cells. Western blot data are representative of three independent experiments. (c) Cell proliferation monitor curves of U87MG/EGFRvIII cells treated with indicated conditions (b). Data represent mean ± s.e.m. (*n* = 3 experiments). ROS (d) and protein carbonyl expression (e) and GSH/GSSG (f) assays found the oxidative stress is decreased after transfection with PINK1, Parkin or both of them in U87EGFRvIII cells. Data represent mean ± s.e.m. (*n* = 3; ^∗^ *P* < 0.05, ^∗∗^ *P* < 0.01; Student's *t*-test).

PINK1 was initially identified in cancer cells as a PTEN-induced protein kinase [29]. It possesses both pro- and antitumorigenesis roles. To delineate its biological relationship with NQO1, we first analyzed their expression patterns in these GBM cell lines by immunoblotting. We found that PINK1 transcription factor FOXO3a was expressed in all of the cells with lower concentrations in both U87MG/EGFRvIII and U87MG/PTEN cells (Figure 6(a), top panel). Consequently, its downstream target PINK1 basically echoed the expression pattern (Figure 6(a), 2^nd^ and 3^rd^ panels). It is worth noting that a similar expression format was found for NQO1 except for its low level in U87MG/EGFR cells. Noticeably, antioxidative enzymes NQO1, catalase, and SOD1 but not SOD2 were all decreased in U87MG/EGFRvIII cells (Figure 6(a)). To test the effect of PINK1 on these antioxidative enzymes, we transfected U87MG/EGFRvIII cell with His-PINK1 or Flag-Parkin or their combination. Overexpression of PINK1 attenuated FOXO3a levels and decreased p-Akt signals without changing total Akt expression levels. We made the similar observations with catalase, NQO1, and SOD1 but not SOD2. Parkin overexpression also reduced expression of these proteins and the peak effect occurred, when both PINK1 and Parkin were mixed together (Figure 6(b)). Presumably, PINK1 and Parkin jointly orchestrate the stabilities these proteins via Parkin-mediated polyubiquitination and subsequent degradation. In a cell proliferation assay, PINK1 or Parkin or their combined overexpression completely suppressed U87MG/EGFRvIII cell growth (Figure 6(c)). Either PINK1 or Parkin overexpression significantly repressed ROS levels, and the maximal effect took place when both of them were cotransfected (Figures 6(d)–6(f)).

## 4. Discussion

In the present study, we demonstrate that activity of the antioxidative enzyme, NQO1, is primarily dictated by the tumor suppressor PTEN in U87MG GBM cells in the presence of wild-type EGFR or the constitutively active EGFRvIII mutant. This effect also applies to LN229 GBM cells regardless of EGFR levels. Overexpression of NQO1 strongly promotes the cell growth of both cell types, especially when EGFR is overexpressed. On the other hand, depletion of NQO1 robustly diminishes the cell proliferation, supporting that NQO1 levels are essential for determining proliferation of these tumor cells. Notably, NQO1 plays a critical role in regulating the ROS levels in the most malignant U87MG/EGFR, EGFRvIII cells but not LN229 GBM cells. NQO1 depletion escalates p-EGFR in U87MG cells without an effect on p-Akt or p-Erks; by contrast, its knockdown does not alter p-EGFR in LN229 cells. Nonetheless, NQO1 depletion reduces both p-Akt and p-Erks in LN229 cells. Hence, this finding suggests that the antioxidative contribution by NQO1 varies in different cell lines, depending on their intrinsic genetic backgrounds.

Currently, it is not clear how PTEN molecularly mediates Nrf2 or NQO1 expression. Because NQO1 levels are tightly coupled with ROS status, it is possible that PTEN might regulate NQO1 expression by altering intracellular ROS levels (Figures 1 and 2). Cancer cells possess increased basal levels of ROS that are necessary for their high proliferative rate. These high levels of ROS result from the increased basal metabolic activity, mitochondrial dysfunction, hypoxia, or mitophagy, as well as enhanced activity of known ROS sources, such as NADPH oxidase [30–32] in cancer cells. In order to eliminate ROS, cancer cells also stimulate the antioxidant system, such as MnSOD, catalase, and glutathione peroxidase [33]. Conversely, ROS provokes intracellular signaling events in tumor cells [34, 35] that lead to cell proliferation. The NQO1 gene is primarily regulated by Keap1/Nrf2/ARE pathway [17, 18], and it can be induced by xenobiotics, antioxidants, oxidants, UV light, and ionizing radiations as well. Kelch-like ECH-associated protein 1 (Keap1) is a chemical and oxidative stress sensor protein that acts as an E3 ligase targeting Nrf2 for degradation [36, 37]. Under the basal condition, Keap1 associates with Nrf2 and induces its ubiquitination and proteasomal degradation. In response to stress, the cysteine residues in Keap1 are modified, leading to Nrf2 nuclear translocation, where it binds to the ARE motif-containing promoter to increase target gene transcription [38]. The MAPK/Erk signaling pathway downstream from EGFR also activates Nrf2-mediated cell proliferation in lung cancer cells [39]. Moreover, nuclear EGFR phosphorylates Keap1 and reduces its nuclear protein level, stabilizing nuclear Nrf2 and increasing its transcriptional activity in cancer cells [40].

Forkhead box, subgroup O (FOXO)3a, belongs to the FOXO subfamily of transcription factors, and its activation usually results in either cell cycle arrest or cell death. FOXO3a protects cells from oxidative stress-mediated apoptosis by increasing MnSOD and peroxisomal catalase [41, 42]. FOXO3a also controls PINK1 transcription in response to growth factor deprivation. Further, FOXO-deficient HSCs (hematopoietic stem cells) exhibit a marked increase in reactive oxygen species (ROS) [43], suggesting a critical role for FOXOs in response to physiologic oxidative stress. Growth factor deprivation activates FOXO3a, leading to upregulation of PINK1 [44]. In addition to FOXO3a, Nrf2 also positively regulates PINK1 expression under oxidative stress conditions [45]. As shown here, PINK1 and Nrf2 expression levels tightly correlate with each other in both U87MG and LN229 GBM cell lines (Figures 2 and 5). Since Nrf2 and PINK1 display a positive sensitivity after the elevation of oxidative stress in the microenvironment surrounding tumors [45], presumably, the upregulation of Nrf2 and PINK1 might be due to an increase in oxidative stress triggered by NQO1 depletion.

PINK1 interacts with the pivotal oncogenic PI3K/Akt/mTOR signaling axis, mediating the critical mitochondrial and metabolic functions that regulate cancer survival, growth, stress resistance, and the cell cycle. PINK possesses a cytoprotective and chemo-resistant function, demonstrating it might be a target in cancer therapeutics [46]. Therefore, it is not surprising that Akt signaling is manipulated, when PINK1 is altered upon NQO1 depletion or overexpression (Figures 2 and 4). In alignment with these observations, it has been reported that PINK1 can both activate Akt and regulate its association with mitochondria powering anaerobic glycolysis or it can be induced when the Akt system is turned off via both FOXO and PTEN [46]. Nevertheless, PINK1 antagonizes cell growth and the Warburg effect in glioblastoma [47]. PINK1 mutation causes an autosomal recessive and early onset PD (Parkinson's disease) [48]. PINK1 possesses an N-terminal mitochondrial targeting sequence and a serine/threonine kinase domain, homologous to calcium/calmodulin-regulated kinase 1 (CAMK1). PINK1 plays a critical role in mediating mitochondrial functions [49, 50]. PINK1 knockdown or expression of kinase-dead PINK1 mutants reduces ATP generation and oxygen consumption [51–53] and increases ROS levels [54]. PINK1 mutations occur in some of cancers, while PINK1 expression is increased in some cancer tissues and decreased in others [55]. The potential pro- and antitumorigenic properties of NQO1 have been well documented. While much current evidence points to PINK1 as a tumor promoter and mediator of chemo resistance, PINK1 possesses a dual role in cancer biology, with context dependent pro- and antitumorigenic properties [55]. Mechanistically, the duality of PINK1 function in cancer cell biology may stem from its regulation of mitophagy, which may be tumor promoting in some circumstances and tumor suppressive in others, depending on the cellular context. Fitting with the dual pro- and antitumorigenesis activities by PINK1, NQO1 also displays the paradoxical effects. For instance, NQO1 acts as an anticancer enzyme, since it protects cells from oxidative stresses through inhibition of quinones from entering the one electron reduction to semiquinone free radicals and ROS [56]. Several types of human cancers are developed due to the NQO1 polymorphisms reducing the NQO1 enzymatic activities [22, 57, 58]. Although a lower or absent NQO1 activity is correlated with increased susceptibility for development of human cancers, NQO1 is overexpressed in numerous human cancers. The high-level expression of NQO1 is associated with lower disease-free survival or 5-year overall survival rates [23]. Conceivably, PTEN may regulate both NQO1 and PINK1 expression in EGFR-amplified GBMs, mediating their antichemotherapy resistance effect. Thus, NQO1 inhibitors might provide innovative pharmacological treatment strategy for therapeutic intervention to the most malignant EGFR amplified gliomas.

## Data Availability

The data used to support the findings of this study are included within the article.

## Supplementary Materials

Supplementary Materials Relative quantification of main secondary metabolites detected in the chórta decoctions. Detection at 280 nm.
