# Supplementary material for: NQO1 Is Regulated by PTEN in Glioblastoma, Mediating Cell Proliferation and Oxidative Stress
Source: Oxid Med Cell Longev. 2018 Nov 25;2018:9146528. doi: 10.1155/2018/9146528 (PMC6286748; doi:10.1155/2018/9146528)
Supplement: Supplementary Materials — Relative quantification of main secondary metabolites detected in the chórta decoctions. Detection at 280 nm. [file 9146528.f1.docx]

Supplementary Materials for

**NQO1 Is Regulated by PTEN in Glioblastoma, Mediating Cell Proliferation and**

**Oxidative Stress**

Shilin Luo^1,2^, Kecheng Lei^1^, Daxiong Xiang^2^ and Keqiang Ye^1,^ ^#^

^1^Department of Pathology and Laboratory Medicine

Emory University School of Medicine

Atlanta, GA 30322

USA

^2^Department of Pharmacy

the Second Xiangya Hospital

Central South University, Changsha, Hunan 410011

China

**Running title:** PTEN mediates NQO1 expression and GBM proliferation

# To whom all correspondence should be addressed (E-mail: [kye@emory.edu)](mailto:kye@emory.edu))

**Table of Contents**

Figure S1. NQO1 expression in GBM cells correlates with different ROS levels.

Figure S2. NQO1 knockdown diminishes U87MG/EGFRvIII/PTEN cell proliferation.

Figure S3. NQO1 overexpression has no effect on the oxidative stress in LN229 GBM cells.

**Figure S1. NQO1 expression in GBM cells correlates with different ROS levels.**

**a** Western blot analysis of EGFR signaling pathways in LN229 and LN229/EGFR GBM cells with EGF stimulation. Cells were incubated with EGF (50 ng/mL) for the indicated time after serum starvation overnight. EGFR signaling pathways were monitored through the indicated antibodies. **b** ROS staining for LN229 GMB cells. ROS-positive cells as detected by an indicator dye CM-H_2_DCFDA. Scale bar, 50 μm. **c** Quantification of ROS intensity **b**. Data represent mean ± s.e.m. (n = 3, **P* < 0.05; student’s t-test). **d** Immunofluorescent co-staining NQO1 and ROS in various U87MG GBM cells. Western blot data and co-staining results are representative of three independent experiments with 3-4 replicates for each experimental condition.

**Figure S2. NQO1 knockdown diminishes U87MG/EGFRvIII/PTEN cell proliferation.**

**a** Cell proliferation assay was conducted for U87MG cells with PTEN expression after transfected with si-control or NQO1 siRNA for up to 4 days. Data represent mean ± s.e.m. (n = 3; **P* < 0.05, ***P* < 0.01; student’s t-test). **b** Western blot analysis of EGFR signaling pathways after NQO1 knockdown. **c** ROS staining by the indicator dye CM-H_2_DCFDA for NQO1 knockdown compared with si-control in U87MG cells with PTEN expression. Scale bar, 50 μm. **d-f** Quantification of ROS positive cells in **c** (mean ± s.e.m.; n = 5; n.s means no statistically significant, **P* < 0.05; student’s t-test). Protein carbonyl expression (**g**) and GSH/GSSG ratio (**h**) analysis after NQO1 knockdown. Data represent mean ± s.e.m. (n = 3; **P* < 0.05; student’s t-test). Western blot data are representative of three independent experiments.


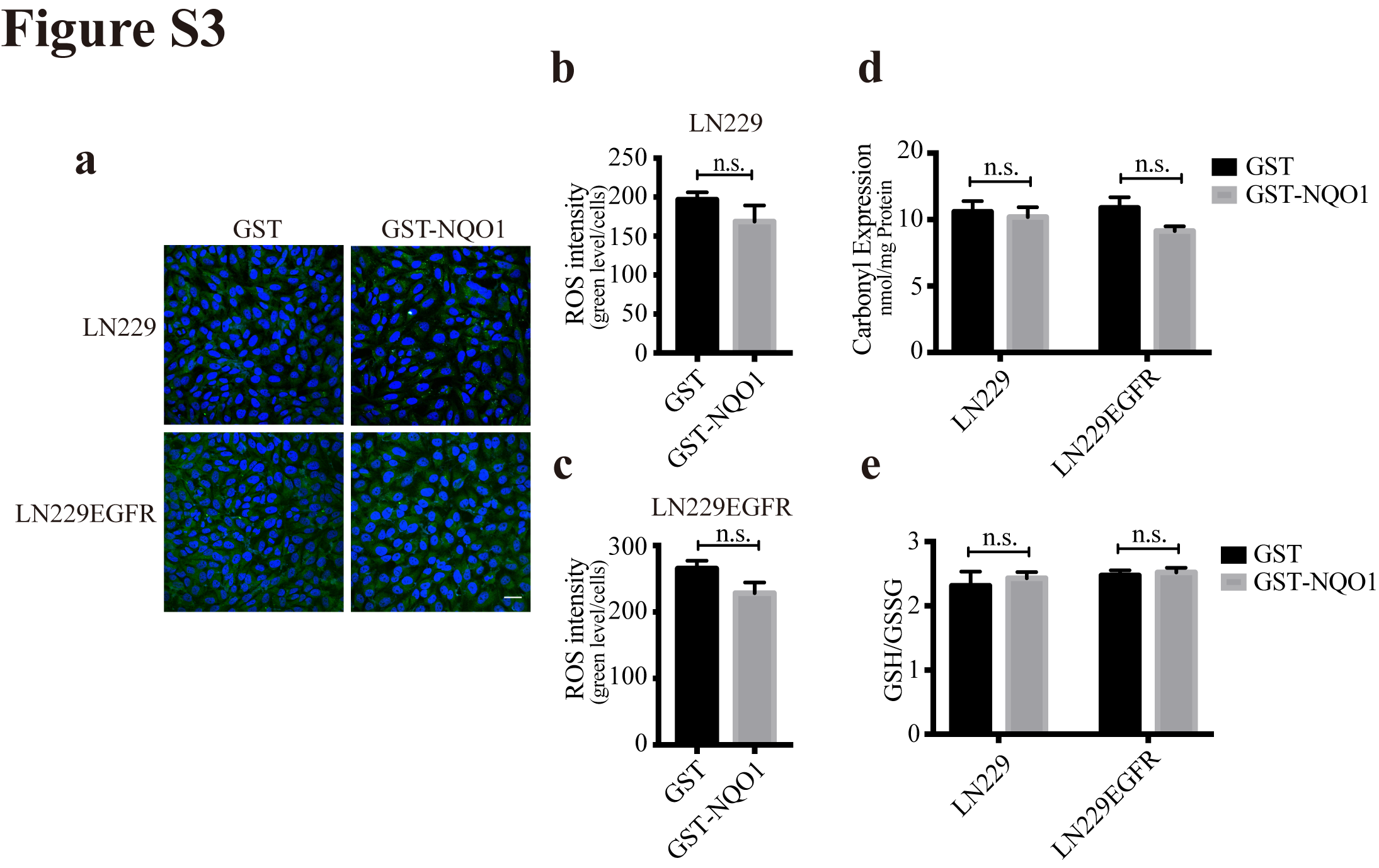


**Figure S3. NQO1 overexpression has no effect on the oxidative stress in LN229 GBM cells.**

**a** ROS staining in LN229 GMB cells after transfection with GST or GST-NQO1. **b** Quantification of ROS intensities **a**. Protein carbonyl expression (**d**) and GSH/GSSG ratio (**e**) analysis for whole cell lysates of LN229 GBM cells transfected with GST or GST-NQO1. Data represent mean ± s.e.m. (n = 3; n.s means no statistically significant; student’s t-test).
